# Variation of rhizosphere bacterial community diversity in the desert ephemeral plant *Ferula sinkiangensis* across environmental gradients

**DOI:** 10.1038/s41598-020-75591-8

**Published:** 2020-10-28

**Authors:** Tao Zhang, Zhongke Wang, Xinhua Lv, Hanli Dang, Li Zhuang

**Affiliations:** College of Life Sciences, Key Laboratory of Xinjiang Phytomedicine Resource Utilization, Ministry of Education, Shihezi University, Shihezi, 832003 Xinjiang China

**Keywords:** Microbiology, Bacteriology, Microbial communities, Environmental microbiology

## Abstract

*Ferula sinkiangensis* (*F. sinkiangensis*) is a desert short-lived medicinal plant, and its number is rapidly decreasing. Rhizosphere microbial community plays an important role in plant growth and adaptability. However, *F. sinkiangensis* rhizosphere bacterial communities and the soil physicochemical factors that drive the bacterial community distribution are currently unclear. On this study, based on high-throughput sequencing, we explored the diversity, structure and composition of *F. sinkiangensis* rhizosphere bacterial communities at different slope positions and soil depths and their correlation with soil physicochemical properties. Our results revealed the heterogeneity and changed trend of *F. sinkiangensis* rhizosphere bacterial community diversity and abundance on slope position and soil depth and found Actinobacteria (25.5%), Acidobacteria (16.9%), Proteobacteria (16.6%), Gemmatimonadetes (11.5%) and Bacteroidetes (5.8%) were the dominant bacterial phyla in *F. sinkiangensis* rhizosphere soil. Among all soil physicochemical variables shown in this study, there was a strong positive correlation between phosphorus (AP) and the diversity of rhizosphere bacterial community in *F. sinkiangensis*. In addition, Soil physicochemical factors jointly explained 24.28% of variation in *F. sinkiangensis* rhizosphere bacterial community structure. Among them, pH largely explained the variation of *F. sinkiangensis* rhizosphere bacterial community structure (5.58%), followed by total salt (TS, 5.21%) and phosphorus (TP, 4.90%).

## Introduction

*F. sinkiangensis* is a short-lived medicinal desert herb, which was included in the pharmacopeia of the People's Republic of China in 1977^1^. Currently, it is only distributed in the yining region of xinjiang, China. *F. sinkiangensis* has important medicinal values, such as anti-cancer^2,3^, antioxidant^4^, antibacterial^5,6^, anti-inflammatory^7^, anti-influenza^5,8^ and anti-diabetes activities^9^. However, high medicinal value of *F. sinkiangensis* leads to the excessive exploitation by people^10^. As a result, the ecological environment of *F. sinkiangensis* habitat is severely damaged, and the population size decreases sharply. In addition, pests^11^ and the low natural reproduction capacity of *F. sinkiangensis* (low seed yield and male sterility)^12^ result in weak population recovery, which leads to a shrinkage of the distribution area of *F. sinkiangensis*, from 1400/ha in 1987 to 133/ha in 2012, and now the number of individuals is still decreasing continuously^13^. Hence, the need to protect *F. sinkiangensis* is imminent.

Soil microbes play a major role in the function of terrestrial ecosystems. This includes decomposition of organic matter, nutrient cycling (carbon, nitrogen and phosphorus) and pollutant conversion^14–17^. It is worth emphasizing that the rhizosphere microbes which are closely related to plants are the most important among these soil microbes. These rhizosphere microorganisms obtain the carbon source and energy needed for survival from plant root exudates and litter^18–21^, but also provide many benefits for plants. For example, regulating the morphology and physiology of plants^22,23^, promote plant growth and damage repair^24–26^, increase plant tolerance to biological and abiotic stresses^27–29^, and improve plant ecological adaptability. Thus, the rhizosphere microbiome is considered as the second genome to plant condition^30^ and serves on a highly evolved external functional environment for plants^31^, which are conducive to plant survival and population recovery. Similarly, we believe that rhizosphere microorganisms can also provide many benefits for the growth and development of *F. sinkiangensis*, which is conducive to the population recovery of *F. sinkiangensis*. Therefore, it is particularly important to understand the structure and composition of soil microorganisms in the rhizosphere of *F. sinkiangensis*. However, our knowledge no scholars have studied the rhizosphere microorganisms of *F. sinkiangensis*, the distribution and structural composition of the rhizosphere soil microbial community of *F. sinkiangensis* are unclear. Therefore, our primary purpose in this study was to explore the structure composition and diversity of the bacterial community in the *F. sinkiangensis* rhizosphere. In addition, *F. sinkiangensis* grows on the hillside and receives different illumination time and intensity at different slope positions^44^. At the same time, the degree of rainwater erosion was different in different slope positions, and the degree of nutrient redistribution was also different in the erosion process^42,43^. Whether the difference caused by slope position will affect the community structure and diversity of bacteria in the rhizosphere soil of *F. sinkiangensis* is not clear, nor has it been reported by other scholars. As far as we know, there are differences in nutrient content in different soil depths, whether these differences will affect the rhizosphere bacterial community of *F. sinkiangensis* is not clear. Therefore, in this study, we investigated the structure composition and diversity of rhizosphere microorganisms in *F. sinkiangensis* under different slope positions and soil depths.

Similarly, researchers have found that environmental factors can directly and indirectly affect soil microbial communities. For example, changing water and nutrient availability can affect root exudates and regulate the rhizosphere microbial diversity^35,36^, soil pH also has a significant effect on the growth of microbial communities^37,38^, nitrogen deposition and precipitation can impact microorganisms^39^ and increase nitrogen significantly affects fungal community, etc. We speculate that some physicochemical factors in the soil would also significantly affect the bacterial community in the rhizosphere soil of *F. sinkiangensis*. But we don't know exactly which factors might affect them. Unfortunately, no other researchers have conducted these studies. Therefore, in this study, we explored the relationship between some physicochemical factors and soil microbes in *F. sinkiangensis* rhizosphere. The purpose is to explore which of these soil physical and chemical factors are significantly related to soil microorganisms in *F. sinkiangensis* rhizosphere.

Giving the spatial heterogeneity of microorganisms^32–34^ and the influence of numerous soil physicochemical factors on microorganisms, we hypothesized that (1) the bacterial community structure and diversity of *F. sinkiangensis* rhizosphere are significantly different at various slope positions and soil depths and (2) there are only a few Soil physicochemical properties having dominant influence on the diversity and structure of the bacterial community in the *F. sinkiangensis* rhizosphere soil.

## Results

### Phylum and genus of dominant bacteria in *F. sinkiangensis* rhizosphere soil

All samples contained 2,360,809 raw tags in total, filtering out the low quality tags and removing the chimeric sequences to obtain the final 2,258,857 effective tags. On average, these tags were grouped into 4546 bacterial Operational Taxonomic Units (OTUs) and contained a total of 76,585 taxon tags per sample (Supplementary Table S1). In addition, the results of the rarefaction curves drawn based on OTUs (Supplementary Figure S1) suggested that the curve representing each soil sample eventually tends to be flat. With the increasing Sequences number, it is rarely accompanied by the increase of the number of new bacteria OTUs, which reflects the reasonable amount of data we sequenced.

**Figure 1:**
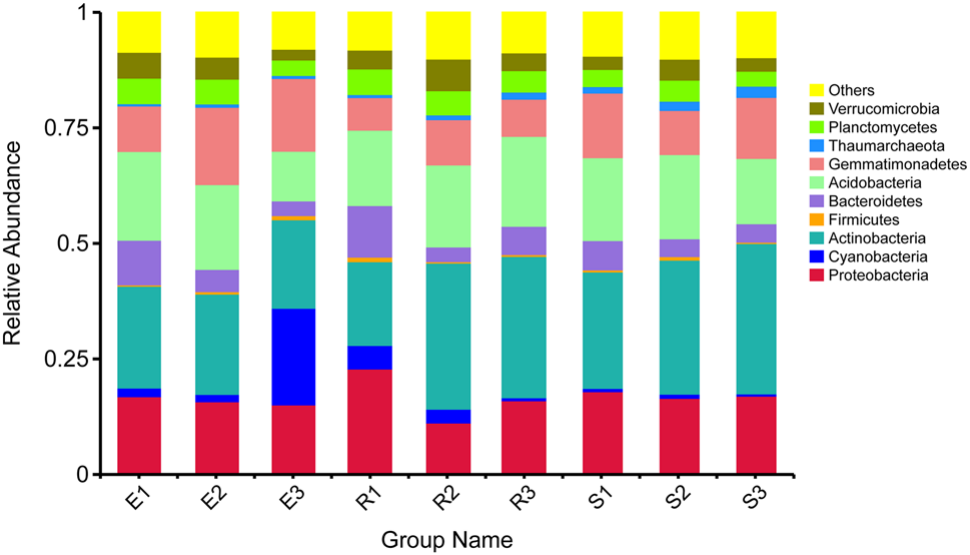
The relative abundance of the top ten phyla in the *F. sinkiangensis* rhizosphere. Distribution histogram is drawn based on R software (Version 2.15.3) ggplot2 package^88^; E, R and S represent the top, middle and bottom of slope respectively; 1, 2 and 3 represent soil depths of 0–10 cm, 10–25 cm and 25–40 cm respectively; Abscissa is the sample name; Ordinate indicates the relative abundance; Other indicate phylum outside the top 10 of the annotated list and the parts not annotated.

Sequence analysis identified 60 phyla and 901 genera of bacteria. Figure 1 showed the 10 bacterial phyla with the greatest abundance in the bacterial community in *F. sinkiangensis* rhizosphere soil. Among them, the total relative abundance of Actinobacteria, Acidobacteria, Proteobacteria, Gemmatimonadetes and Bacteroidetes was 71.30%, which occupies a dominant position in the rhizosphere soil bacterial community of *F. sinkiangensis*. In addition, supplementary figure S2 showed the 30 bacterial genera with the highest abundance in the bacterial community in *F. sinkiangensis* rhizosphere soil. The sum of their relative abundance was 16.20% and the remaining 83.80% include genera outside the top 30 of the annotated list and the parts not annotated.

### Relationships between slope position and soil depth and dominant rhizosphere bacteria

**Figure 2:**
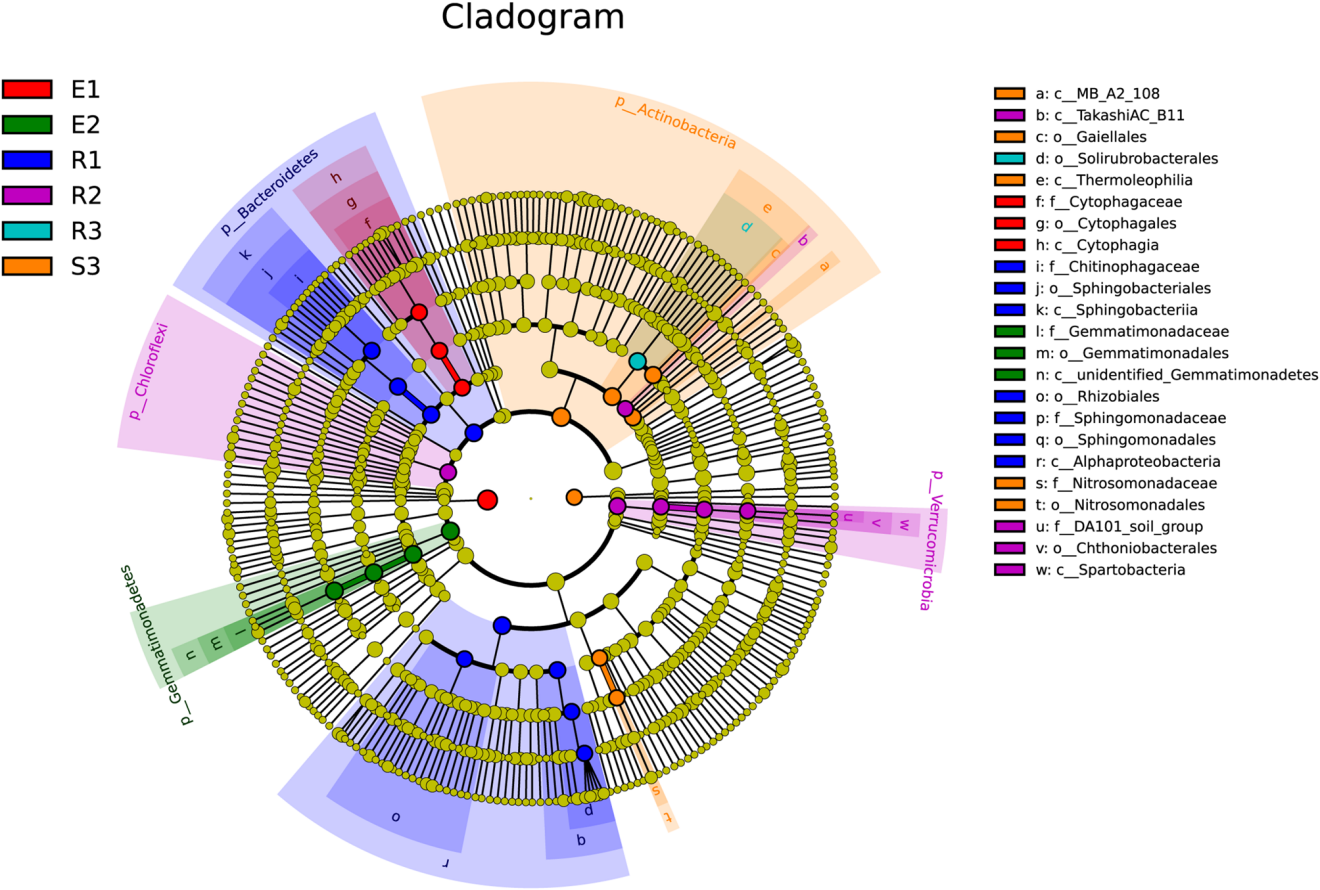
Results of intergroup LDA effect size (LEfSe) analysis revealed the specific bacteria phylum and genus distributed in the *F. sinkiangensis* rhizosphere under different slope positions and soil depths. LEfSe analysis diagram drawn based on LEfSe online software (https://huttenhower.sph.harvard.edu/galaxy/)^84^; In cladograms, the circle radiating from inside to outside represents the classification level from the Phylum to the species. Each small circle at different classification levels represents a classification at that level, and the diameter of the small circle is proportional to the relative abundance; Species with no significant differences are uniformly colored in yellow; Different species Biomarker follows the group for coloring.

Two-way analysis of variance and LDA effect size analysis showed that the relative abundance of Proteobacteria, Cyanobacteria, Actinobacteria, Firmicutes, Bacteroidetes, Gemmatimonadetes, Thaumarchaeota and Verrucomicrobia were significantly affected by the slope position (*p* < 0.05), and showed different levels in different soil depths. In the 0–10 cm soil layer, the relative abundance of Proteobacteria, Cyanobacteria, Firmicutes and Bacteroidetes in the middle of the slope was significantly higher than the top and bottom of the slope. However, the relative abundance of Actinobacteria and Gemmatimonadetes at the top and bottom of the slope was significantly higher than that of the mid-slope. In the 10–25 cm soil layer, the relative abundance of Cyanobacteria and Verrucomicrobia at the mid-slope was significantly higher than the top and bottom of the slope. Interestingly, the relative abundance of Firmicutes in the 10–25 cm soil layer showed a trend of increasing as the slope position decreases, while the opposite was true in the 25–40 cm soil layer (Fig. 2 and Supplementary Table S2). In addition, the results of the two-factor analysis of variance (Table 1) based on the diversity indexes of each sample showed that the Chao1 and Shannon indexes, which evaluate the richness and diversity of bacterial communities in the rhizosphere soil of *F. sinkiangensis*, have significant differences between the main effect soil depth and slope position(*p* < 0.05). At the same time, the interactive effect of soil depth and slope position has no significant effect on the shannon and chao1 indexes. Specifically, the bacteria diversity and richness at the top of the slope were higher than that at the bottom of the slope; Diversity and richness of bacterial community in the soil layer of 0–10 cm was significantly higher than that in the soil layer of 10–25 cm and 25–40 cm.

**Table 1:** Inter Subject Effect test of the impact of slope position and soil depth on Shannon and Chao1 index.

	Dependent variable	Type III sums of squares	Degree of freedom	Mean square	F	Statistical significance
Model modification	Shannon index	37.319^a^	8	4.665	2.905	0.029
	Chao1 index	31,700,036.6^b^	8	3,962,504.580	6.491	0.000
Intercept	Shannon index	1991.162	1	1991.162	1240.048	0.000
	Chao1 index	393,690,702.7	1	393,690,702.7	644.892	0.000
Soil depth	Shannon index	14.882	2	7.441	4.634	0.024
	Chao1 index	11,511,392.39	2	5,755,696.197	9.428	0.002
Slope position	Shannon index	16.611	2	8.306	5.173	0.017
	Chao1 index	13,356,100.35	2	6,678,050.176	10.939	0.001
Soil depth * Slope position	Shannon index	5.826	4	1.457	0.907	0.481
	Chao1 index	6,832,543.892	4	1,708,135.973	2.798	0.057

Two-factor analysis was drawn based on SPSS (SPSS 19.0; IBM Inc., Armonk, USA)^85^.

### Relationship between slope position, soil depth and *F. sinkiangensis* rhizosphere soil physicochemical properties

Spearman correlation analysis showed that the available phosphorus (AP) was significantly positively correlated with the total organic carbon (TOC), total nitrogen (TN) and total potassium (TK); TOC was significantly positively correlated with TN, AP and total salt (TS); Ammonium nitrogen (AN) was negatively correlated with the total phosphorus (TP) ; There was a significant negative correlation between pH and TS. However, there was no significant correlation between most soil physicochemical variables (Table 2). In addition, multivariate analysis of variance showed that slope position was significantly correlated with most soil physicochemical properties including TOC, TP, TK, NN, AN, AP, and TS. However, only a few soil physicochemical properties, including AP, TS and pH, had a significant correlation with depth. Specifically, TOC, TP, TK, AP, and TS were significantly higher at the top than the middle of slope. The contents of TP and AP at the top of slope were significantly higher than those at the bottom and middle of slope. Interestingly, the content of AN was significantly higher at the bottom than at the top and middle of slope. In terms of depth, the content of AP and pH in 0–10 cm soil were significantly higher than those in 25–40 cm soil (Supplementary Table S3).

**Table 2:** Spearman correlation coefficients between soil physicochemical properties across all samples.

	TOC	TN	TP	TK	NN	AN	AP	TS	pH
TOC	1.000								
TN	0.792**	1.000							
TP	0.163	0.193	1.000						
TK	0.171	0.333	0.067	1.000					
NN	0.356	0.277	− 0.149	− 0.079	1.000				
AN	− 0.200	− 0.269	− 0.448*	0.012	0.031	1.000			
AP	0.568**	0.639**	0.271	0.472*	0.069	− 0.230	1.000		
TS	0.411*	0.232	0.004	0.125	0.287	− 0.031	0.073	1.000	
pH	0.038	0.149	0.002	0.129	0.077	0.047	0.213	− 0.390*	1.000

Spearman correlation table is drawn based on SPSS (SPSS 19.0; IBM Inc., Armonk, USA)^85^.

*TS* total salt content, *AP* available phosphorus content, *AN* ammonium nitrogen content, *NN* nitrate nitrogen content, *TP* total phosphorus content, *TN* total nitrogen content, *TK* total potassium content, *TOC* total organic carbon content.

r-value represents Spearman correlation coefficient, between − 1 and 1, r < 0 is negative correlation, r > 0 is positive correlation *p < 0.05, **p < 0.01.

### pH largely explained the variation of *F. sinkiangensis* rhizosphere bacterial community structure

**Figure 3:**
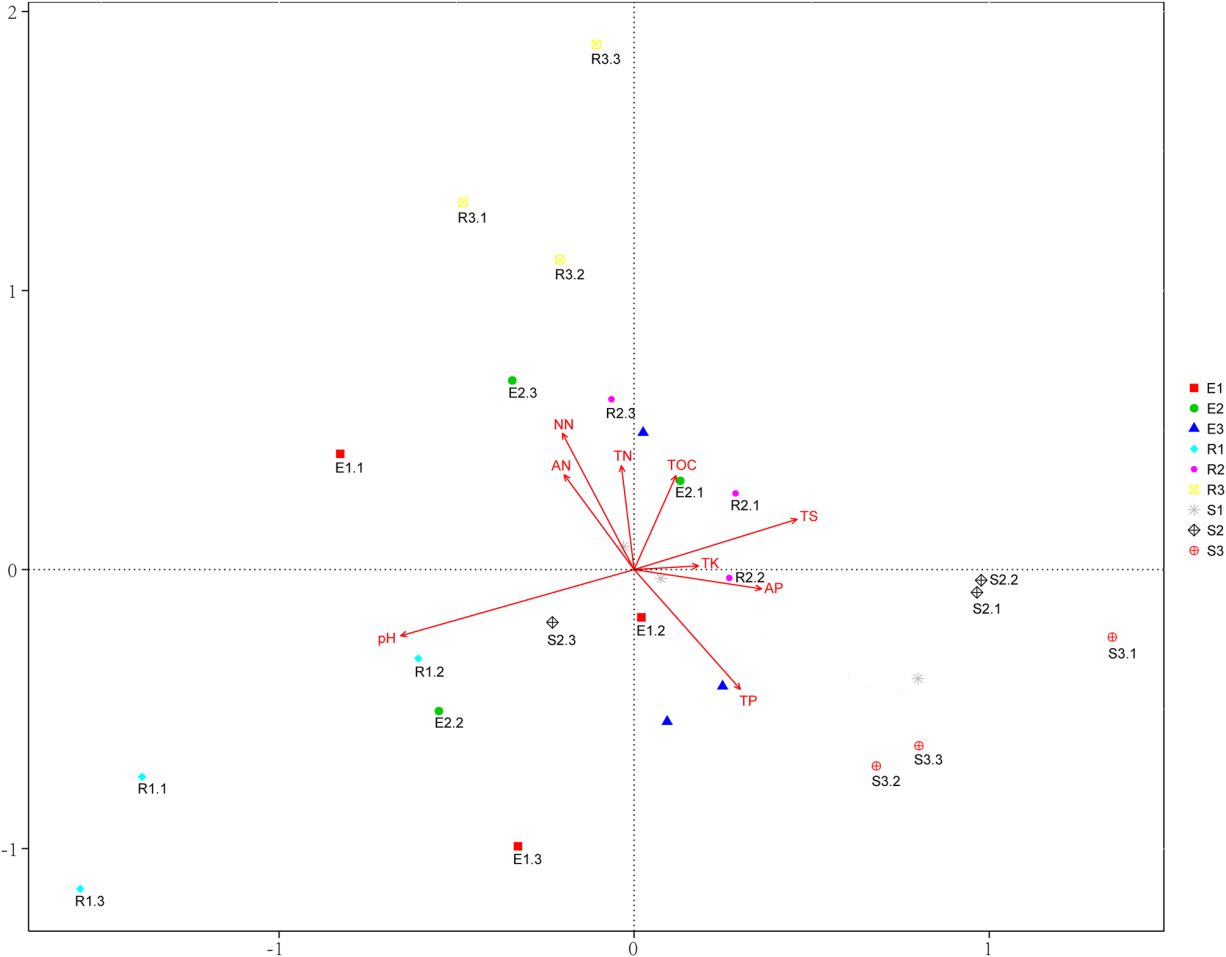
Ordination diagram (samples-environment biplot) of db-RDA depicting environmental drivers of rhizosphere bacterial community composition of *F. sinkiangensis*. db-RDA figure is drawn based on Canoco Software (Version 5.0)^87^; E, R and S represent the rhizosphere region of the top, middle and bottom of the slope respectively; Numbers indicate root depths, 1, 2 and 3 representing depths of 0–10 cm, 10–25 cm and 25–40 cm, respectively.

Across all samples, soil AP showed a significant positive correlation with α-diversity of the bacterial community (r = 0.538, p < 0.01). Soil TP and TK and the α-diversity displayed a significant positive correlation (Shannon index, r = 0.495 and 0.405, respectively, *p* < 0.05, Table 3). In addition, distance-based redundancy analysis (db-RDA) also showed a correlation between soil physicochemical properties and the distribution of bacterial communities in the rhizosphere of *F. sinkiangensis* (Fig. 3). All the soil physicochemical factors explained 24.28% of the variation in the rhizosphere bacterial community structure of *F. sinkiangensis*. The pH explained 5.58% of variation, TS 5.21%, TP 4.90%, NN 3.89% and AP explained 3.60%. Among them, pH, TS and TP explained the largest proportion of the variation of the bacterial community structure in the rhizosphere of *F. sinkiangensis* (Supplementary Table S4).

**Table 3:** The spearman correlation analyses between the α-diversity index and soil physicochemical properties.

	Shannon index		Chao1 index	
	R	*p*	r	*P*
TOC	0.234	0.240	− 0.037	0.854
TN	0.185	0.356	− 0.152	0.449
TP	0.459	0.016*	0.114	0.571
TK	0.405	0.036*	0.225	0.259
NN	0.060	0.766	− 0.102	0.613
AN	− 0.312	0.113	0.078	0.698
AP	0.538	0.004**	0.240	0.228
TS	0.027	0.892	− 0.035	0.862
pH	0.317	0.498	− 0.190	0.343

Spearman correlation table is drawn based on SPSS (SPSS 19.0; IBM Inc., Armonk, USA)^85^.

r-value represents spearman correlation coefficient, between − 1 and 1, r < 0 is negative correlation, r > 0 is positive correlation *p < 0.05, **p < 0.01.

### Spearman correlation analysis between relative abundance of dominant bacteria (phyla and genera) and soil physicochemical properties

**Figure 4:**
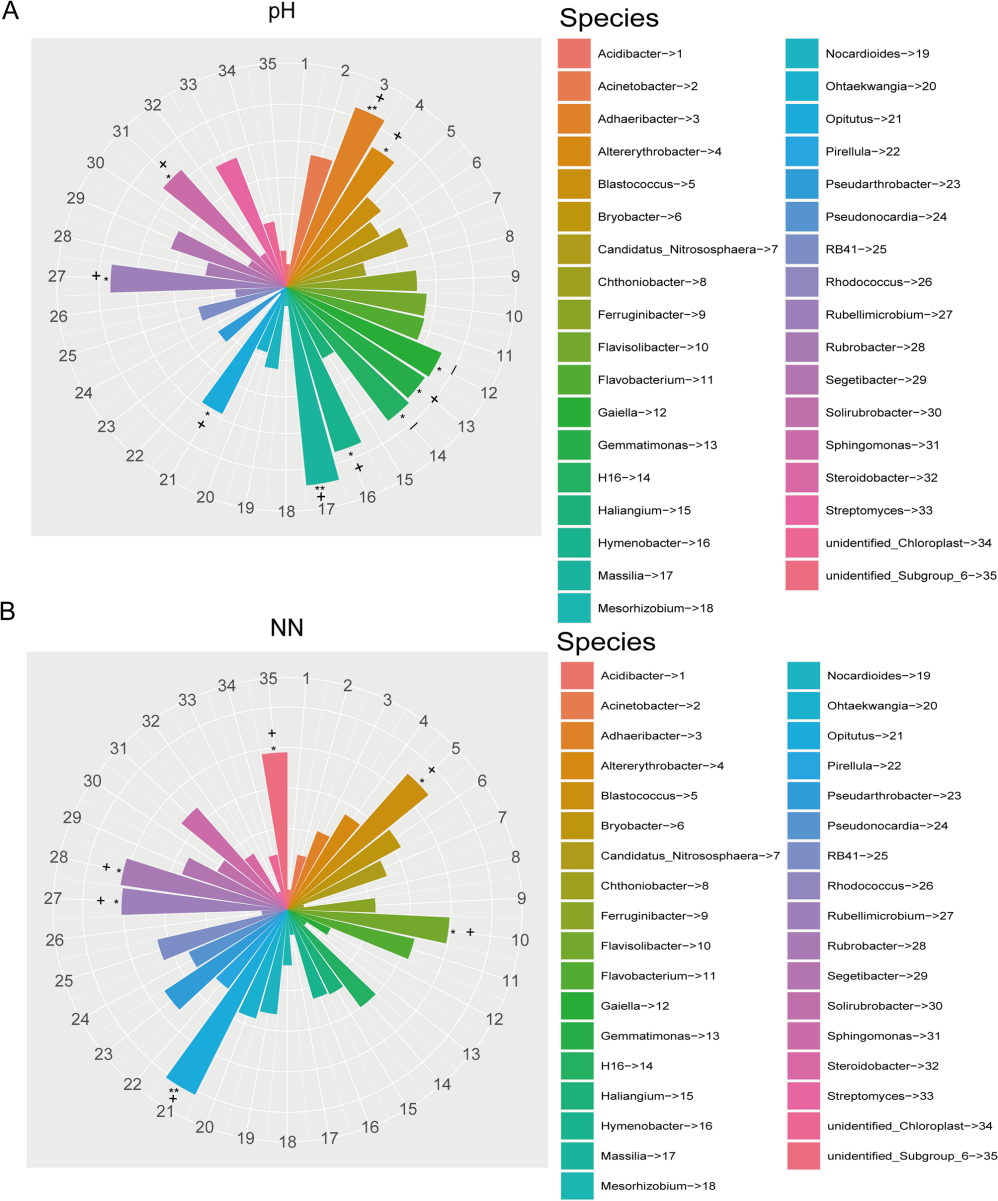
The spearman correlation between pH (**A**), NN (**B**) and dominant bacteria. Spearman correlation figure was drawn based on R Software (Version 2.15.3)^86^; “+ ” indicates positive correlation; “−” indicates negative correlation *p < 0.05, **p < 0.01.

Table 4 showed the relationship between the top ten dominant bacteria phyla and soil physicochemical properties. Specifically, TN, AP and TS have a significant positive correlation with the relative abundance of Actinobacteria; AP and TS showed significant positive relationships with Chloroflexi; pH showed significant positive relationships with Bacteroidetes; AN has significant positive relationships with Verrucomicrobia. Conversely, NN and TS significantly negative correlated with the relative abundances of Gemmatimonadetes; TN and AP showed significant negative relationships with Cyanobacteria. Interestingly, Firmicutes, Proteobacteria, Planctomycetes and Acidobacteria were not significantly related to any variables (Table 4). Moreover, the relationship between the relative abundance of the first thirty-five bacterial phyla and the physicochemical properties of the soil was shown in supplementary Fig. S3. In addition, correlation analysis between relative abundance of bacteria genera and soil physicochemical properties showed that pH and NN were significantly correlated with most bacteria genera. For example, pH has significant positive correlation with the relative abundance of *Adhaeribacter*, *Altererythrobacter*, *Gemmatimonas*, *Hymenobacter*, *Massilia*, *Opitutus*, *Rubellimicrobium* and *Sphingomonas* (Fig. 4A). NN has significant positive correlation with *Blastococcus*, O*pitutus*, *Flavisolibacter*, *Rubrobacter* and *Rubellimicrobium* (Fig. 4B).

**Table 4:** The spearman correlation analysis between relative abundance of dominant bacteria and soil physicochemical properties.

	Actinobacteria	Firmicutes	Proteobacteria	Bacteroidetes	Planctomycetes	Acidobacteria	Gemmatimonadetes	Chloroflexi	Verrucomicrobia	Cyanobacteria
pH	− 0.355	− 0.003	0.054	0.434*	0.106	0.13	0.027	− 0.263	0.183	0.21
TOC	0.346	− 0.026	− 0.101	0.049	0.026	0.026	− 0.377	0.311	− 0.049	− 0.291
TN	0.420*	− 0.122	0.087	− 0.035	− 0.224	− 0.096	− 0.151	0.349	− 0.035	− 0.384*
TP	− 0.026	− 0.279	0.314	0.278	0.012	0.12	0.178	− 0.065	− 0.125	− 0.12
TK	0.246	− 0.114	0.026	0.071	− 0.006	0.3	− 0.007	0.337	− 0.089	− 0.302
NN	0.018	− 0.083	− 0.004	0.351	− 0.04	0.028	− 0.564**	0.007	0.094	0.123
AN	0.226	− 0.024	− 0.162	− 0.137	0.174	0.049	0.067	0.196	0.395*	0.073
AP	0.445*	− 0.206	0.187	0.201	− 0.156	− 0.002	− 0.012	0.411*	− 0.123	− 0.492**
TS	0.443*	− 0.169	− 0.238	− 0.206	− 0.09	0.027	− 0.394*	0.394*	0.031	− 0.119

Spearman correlation table is drawn based on SPSS (SPSS 19.0; IBM Inc., Armonk, USA)^85^.

r-value represents spearman correlation coefficient, between − 1 and 1, r < 0 is negative correlation, r > 0 is positive correlation *p < 0.05, **p < 0.01.

## Discussion

One noteworthy result of our study was that the diversity and richness of the bacterial community in the rhizosphere of *F. sinkiangensis* were significantly different at different slope positions and soil depths (Table 1). This result supported our first hypothesis on the bacterial diversity and abundance of rhizosphere bacteria in *F. sinkiangensis* being sensitive to slope and soil depth variation; importantly, the spatial resolution of bacterial communities was detectable on a small spatial scale; These found were consistent with the results of Qi Sun et al.^40^ in exploring the microbial diversity of steep slope soils in the semi-arid Loess Plateau and also Martina et al.^41^ who explored the small-scale spatial diversity of temperate forests.

The variation in diversity and richness of bacterial communities along the slope might be attributed mainly to two aspects. The first aspect was redistribution of nutrients down the slope during rainfall erosion^42,43^, causing differences in soil properties (Supplementary Table S3). As far as we know, there were a significant correlation between soil physicochemical properties and bacterial communities. For example, TN, AP, AN, NN and TS were significantly correlated with dominant bacteria phyla in *F. sinkiangensis* rhizospherical soil (Table 4). NN and pH were significantly correlated with most bacterial genera (Fig. 4). Meanwhile, other physicochemical factors were also significantly correlated with dominant bacterial genera (Supplementary Fig. S3). This means that the diversity and composition of bacterial community in *F. sinkiangensis* rhizosphere soil were greatly different, which may be caused by the different distribution of soil physicochemical properties. The second aspect was the illumination time and intensity different at various slope positions^44^. For example, there was a longer illumination time at the top of the slope than at the bottom, which was conducive to increased photosynthetic formation of organic compounds by plants, thus increasing the input of TOC into soil. As far as we know, there were significant correlations between TOC and some other physicochemical properties in soil. For example, there was a significant positive correlation between TOC and TN, AP and TS (Table 2). This means that the difference of TOC input can lead to the difference of other soil physicochemical properties, and finally affect the structure composition and diversity of the bacterial community in the soil.

Stratification differences in the diversity and richness of bacterial communities with soil depth (Table 1) were similar to those in the studies of Fierer et al. and Eilers et al.^45,46^ mainly due to spatial heterogeneity of nutrients (Supplementary Table S3). The topsoil has greater contents of nutrients than the deeper soil layers, with a consequent increase in microbial α-diversity^47^. Secondly, the topsoil microorganisms have higher activity than the deep soil microorganisms. Microbial decomposition and metabolic processes result in a large number of metabolites (including extracellular enzymes), contributing to topsoil nutrient accumulation and thus growth of bacteria.

Soil pH is considered as the best predictor of bacterial community structure and diversity at different spatial scales and soil types^48–50^, and this was consistent with the results we found in this study regarding the composition and variation of the rhizosphere bacterial community in the rhizosphere of *F. sinkiangensis* (Fig. 3 and Supplementary S4). However, the mechanism behind this model is little known, and we propose three relevant hypotheses. 1. Bacteria have a certain range of tolerance to pH, beyond which they cannot survive. The intracellular pH of many microorganisms is close to neutral^51^. Therefore, any degree of pH change exerts pressure on some bacterial groups with narrow pH tolerance and inadaptability, interfering with their growth, affecting their competitiveness^52^, and eventually leading to changes in the composition of bacterial communities. 2. The pH may not directly affect the structure of bacterial communities, but it may affect the physicochemical properties of soils directly and indirectly. For example, there was a significant negative correlation between pH and salt content (Table 2); in addition, pH affects the availability of nutrients and heavy metal ions in soil^53,54^. Therefore, pH can be used as an integrated variable to combine with other factors in influencing the community structure of bacteria. 3. Singh et al. showed that some plants actively affect soil bacterial communities by changing soil pH through root exudates^55^. In addition, studies have shown that plant growth was passively influenced by pH, regulating the amount and composition of root secretions^54,56^, which further influences nutrient availability, enzyme activity and microbial abundance^57,58^. Of course, pH, whether actively or passively, plays an important role in influencing the structure of bacterial communities.

Interestingly, we found in this study there was a significant correlation between AP (but not pH) and *F. sinkiangensis* rhizosphere bacterial diversity. This suggested that pH was not always a universal predictor of bacterial community diversity. Depending on habitat environment and soil type, there might be factors that explain bacterial diversity better than pH. The reason why AP has a significant relationship with bacterial community diversity was a relationship between C mineralization and P availability. Previous studies showed that in the absence of P, the retention time of litter was longer and the mineralization of unstable C was inhibited. This even had a negative effect on the carbon sequestration in cultivated lands^58,59^. However, C is the main source of energy for microorganisms and interferes with their growth^60^. The response of C mineralization to P availability directly impacts the biodiversity.

Microorganisms can be closely involved in soil nutrient cycle, improved soil nutrient status and optimized soil structure^60–62^. Provide suitable soil environment for plant growth. Considering the important role of microorganisms in plant growth and development, Enkatachalam et al. put forward the "Belowground Solutions to an Aboveground Problem" viewpoint^63^. That is, by trying to solve some of the problems faced by plants on the ground by relying on microbes in the soil below. Meanwhile, the research of some scholars also confirmed this viewpoint. For example, Hoflich et al. stimulated plant growth by inoculation with symbiotic and associative rhizosphere microorganisms^64^, and the microorganisms discovered by Horace et al. regulated plant growth by producing various secondary metabolites^65^. In addition, some scholars applied phosphate, potash and nitrogen fertilizers and organic matter to alter the microbial community diversity and influence plant growth^66,67^. Therefore, we hope that the discovery of pH, AP, TP and TK significantly influencing the bacterial community diversity and structure in the rhizosphere of *F. sinkiangensis* (Table 3 and Supplementary Table S4) can be applied in conservation and commercial cultivation of *F. sinkiangensis*.

## Conclusion

Although *F. sinkiangensis* population continues to decline, the research conducted on *F. sinkiangensis* is still focused mainly on medicinal properties; in contrast, the research on habitat ecology, plant physiology and soil is rare, restricting the capacity to conserve *F. sinkiangensis*. In this study, we explored the diversity and structural composition of the rhizosphere bacterial community in the rhizosphere of *F. sinkiangensis*. However, bacterial communities are made up of a wide variety of bacteria, some of which were reported in other plant–microbe interaction patterns as beneficial to plant growth and development, some of which are neutral, and some of which are not conducive to plant growth and development. This requires us to conduct further characterization in subsequent studies, to identify the bacterial populations conducive to the growth and development of *F. sinkiangensis*.

The spatial heterogeneity of microorganisms has been shown, but there are few studies on sloping land and soil depth. Our study revealed trends in bacterial community diversity at different slopes and soil depths, which will contribute to our understanding of the microbially-mediated carbon cycle in soil. Our study demonstrated the universality of pH prediction of bacterial community structure, but also showed that pH effects on bacterial community diversity might be limited under the influence of specific habitats and soil types. In addition, the dominant effects of pH, AP, TK and TP on the bacterial community were identified, and we considered using them in the conservation and commercial culture of *F. sinkiangensis*. By adjusting pH value and applying phosphate and potassium fertilizers, the diversity and structure of bacterial community might be affected, and soil structure might be improved to provide suitable soil environment for the growth of *F. sinkiangensis*. Exploring the optimal amount and proportion of fertilizers needs to be done in the future studies.

## Materials and methods

### Site description and sampling

Since *F. sinkiangensis* is a short-lived plant, we chose to study it during its growing season (April–May). We set the place of Byshidun, Yili, Xinjiang, China as the research plot (desert gray calcareous soil, slope: 43°, longitude: 82.083359, latitude: 43.723121). On the slopes where *F. sinkiangensis* grew, we designed rectangular plots of 10 m × 5 m at the top, middle and bottom of the slope respectively. Riley and Barber shaking methods^68^ were used to collect rhizosphere soil samples in 10 m × 5 m plots circled at the top, middle and bottom of the slope. Taking the slope top as an example, 3 *F. sinkiangensis* with the same growth potential were randomly selected in the sample plot of 10 m × 5 m. Then the rhizosphere soil samples were collected by shaking^68^ at the roots of 0–10 cm, 10–25 cm and 25–40 cm of each *F. sinkiangensis*. Collected soil was immediately placed in a 5 ml test tube with sterile tweezers, and then marked on a tube and placed in a liquid nitrogen tank for storage. A total of 9 soil samples were collected at the top of the slope. Specifically, 3 depths (0–10 cm, 10–25 cm and 25–40 cm) × 3 replicates. We explored three different plots of top, middle and bottom, with a total of 27 soil samples (3 × 9). In addition, we used a mixed labeling system to label the samples. The first group of letters was used to represent the position of the slope, rhizosphere (E, R and S represent the rhizosphere region of the top, middle and bottom of the slope respectively). The second number indicates the depth (1, 2, and 3 represent 0–10 cm, 10–25 cm, and 25–40 cm depth, respectively) and the third number indicates the number of repetitions. For example, S2.1 represents the first replicate soil sample with a root depth of 10–25 cm in the roots of *F. sinkiangensis* at the bottom of the slope (Supplementary Table S5).

### Soil analysis

Obtained soil samples were naturally air-dried, sieved to 2 mm, and plant impurities were removed for determining physical and chemical properties. Organic matter contents were determined by wet digestion^69^. Nitrogen content was determined by the perchlorate-sulfuric acid digestion method (1035 automatic nitrogen determination apparatus, FOSS)^70^. Total phosphorus contents were assayed by the molybdenum method (Cary 60 UV–Vis spectrophotometer, Agilent)^70^. Total potassium was measured using the acid dissolution—atomic absorption method (Atomic Absorption Spectrometer, Thermo Fisher Scientific). pH measurements were performed with pH-meter (FiveEasy Plus, Mettler-Toledo). Available phosphorus was determined by molybdenum inverse colorimetry after extraction with sodium bicarbonate. Nitrate and ammonium nitrogen was determined by the 0.01 M calcium chloride extraction method (Flow analyzer, BRAN + LUEBBE). Total salt was determined by using dry-residue method (Supplementary Table S6).

### DNA extraction, amplification and library generation

DNA was isolated from each sample using a Centrifugal Soil Genomic DNA Extraction Kit, and the purity and concentration of the DNA were measured by 1% agarose gel electrophoresis. An appropriate amount of DNA sample was placed in a sterile centrifuge tube and diluted to 1 ng/μL with sterile water. Partial 16S rDNA-based high-throughput sequencing was used to determine the bacterial diversity and community composition according to Caporaso et al^71^. Using diluted DNA as a template, PCR amplification of the V4 region of 16S rRNA gene was performed using barcode-specific primers 806R (5′ -GGACTACHVGGGTWTCTAAT-3′) and 515F (5′-GTGCCAGCMGCCGCGGTAA-3′). The V3-V4 region was used because the primers 515F-806R retrieve the greatest diversity of the bacterial domain at the phylum level^72^. Phusion High-fidelity PCR Master Mix with GC buffer from New England Biolabs was used to ensure the efficiency and accuracy of amplification. PCR products were detected by electrophoresis with 2% agarose gel, and target bands between 200 and 300 bp were cut for further experiments. PCR products were mixed at equal density ratios. The PCR products were purified using Qiagen Gel Extraction Kits (Qiagen, Germany). The recovered purified products were placed in a 1.5 mL sterile centrifugal tube in a dry ice box and sent to Beijing Compass Biotechnology Co., Ltd for high-throughput sequencing. The library was constructed using a TruSeq DNA PCR-Free Sample Preparation Kit. The constructed library was quantified by Qubit 2.0 Fluorometer (Termo Scientifc) and Q-PCR (Thermo Fisher Scientific). After the library was qualified, it was sequenced using HiSeq2500 PE250.

### Sequence analysis

After barcode sequences and primer sequences were cut off, the tags of each sample were spliced with a fast and accurate analysis tool FLASH^73^ to obtain the raw tags. Raw tags were carefully filtered^74^ to get clean tags of high quality using Qiime (V1.9.1)^75^. Tags quality control process comprised the following operations: (a) Tags intercept: raw tags with low quality value (default quality threshold was ≤ 19) of the set length (default length value was 3) were truncated; (b) Tags length filtering: the tagged data set obtained after interception of tags was further filtered out of the continuous high quality tags whose base length was less than 75% of the tags length. The tag sequences obtained after the above treatment are compared with the Gold database by UCHIME Algorithm^76^ to detect chimeric sequences that were removed^77^, to obtain the final effective tags.

Uparse software (Uparse v7.0.1001)^78^ was used to cluster the effective tags of all samples, with 97% sequence identity. Grouping sequences into Operational Taxonomic Units (OTUs). The sequences with the highest occurrence frequency in OTUs were selected as representative of OTUs by using the Mothur method. SILVA^79^ SSUrRNA database^80^ was used for OTUs annotation to represent species sequence analysis (setting threshold of 0.8–1) and acquire information on taxonomy. The MUSCLE (Version 3.8.31) software^81^ was used for fast multi-sequence alignment to obtain the system relationship of all OTUs representative sequences. Finally, the sample with the smallest amount of data was homogenized. Subsequent alpha diversity (α-diversity) and beta diversity (β-diversity) analyses were both based on the homogenized data. The raw sequence tags have been deposited in the NCBI Sequence Read Archive (SRA) with BioProject accession number PRJNA667321 (https://dataview.ncbi.nlm.nih.gov/object/PRJNA667321?reviewer=npa1ot26ika639ulfdgqid7p6u).

### Data analysis

QIIME software (Version 1.9.1) was used to calculate the Observed-species, Chao1, Shannon, and Goods-coverage indices. Chao1, Shannon and Goods-coverage indices can be used to evaluate the richness, diversity and sequencing depth of samples respectively^82^. In addition, R software (Version 2.15.3) was used to draw the dilution curve to reflect the rationality of sequencing data volume. β-diversity analysis was performed on weighted and unweighted unifac with the QIIME software (version 1.7.0)^83^. The latter software was used as a hierarchical clustering method to interpret the distance matrix using average link, and arithmetic mean (UPGMA) clustering was used for the unweighted pair method. The linear discriminant analysis (LDA) effect size (LEfSe)^84^ was used to identify statistically significant differences between groups.

Two-way ANOVA (SPSS 19.0; IBM Inc., Armonk, USA)^85^ was used to assess differences in bacterial relative abundance or diversity between samples. Spearman correlation analysis^86^ was performed to assess the interrelationship between soil properties and the dominant bacterial species. In addition, distance-based redundancy analysis (db-RDA) and variance partitions were performed to study the relationship between the composition of the rhizosphere, soil properties and non-soil parameters. This was carried out with the Canoco statistical software (version 5.0) and default parameters (vegan package)^87^.

## Supplementary information

Supplementary Information

## Data Availability

We declare that all experimental data involved in the manuscript have been added to the manuscript and supplementary materials. If readers need further data related to the manuscript, please feel free to contact our corresponding author at any time.
