# Supplementary material for: Variation of rhizosphere bacterial community diversity in the desert ephemeral plant Ferula sinkiangensis across environmental gradients
Source: Sci Rep. 2020 Oct 28;10:18442. doi: 10.1038/s41598-020-75591-8 (PMC7595108; doi:10.1038/s41598-020-75591-8)
Supplement: Supplementary file 1 — Supplementary Information [file 41598_2020_75591_MOESM1_ESM.pdf]

Variation of rhizosphere bacterial community diversity in the desert ephemeral plant *Ferula sinkiangensis* across environmental gradients

Tao Zhang[1], Zhongke Wang[1], Xinhua Lv[1]Hanli Dang[1*]and Li Zhuang[1*]

[1] College of Life Sciences, Key Laboratory of Xinjiang Phytomedicine Resource Utilization, Ministry of Education, Shihezi University, Xinjiang Shihezi, 832003, China.

* Correspondence:Hanli Dangemail: 3497404295@qq.com; Li Zhuang email:3033573705@qq.com

Supplementary Table (Table S1-6) and supplementary Figure (Figure S1-3)

Table S1 Statistics of quality control of the barcoded Illumina HiSeq 2500 tags results.

Sample	Raw tags	Effective Tags	Effective tags base	Effective tags length	Taxon tags	OUT number	GC content (%)	Effective (%)
E1.1	89,407	85,046	21,555,116	253	79,873	4,777	57.13	91.57
E1.2	90,218	86,348	21,853,033	253	78,123	4,591	57.48	92.63
E1.3	81,003	77,632	19,686,444	254	73,803	4,839	56.88	92.55
E2.1	85,356	81,158	20,547,290	253	70,271	4,252	58.20	91.23
E2.2	91,758	87,766	22,227,035	253	82,280	4,696	57.64	92.44
E2.3	76,687	72,596	18,385,757	253	64,417	4,185	58.11	90.64
E3.1	80,441	76,031	19,290,268	254	66,861	3,980	58.56	91.22
E3.2	84,291	81,047	20,493,293	253	76,731	3,375	56.48	92.90
E3.3	91,051	87,447	22,215,695	254	82,704	4,714	57.98	92.33
R1.1	94,093	90,000	22,775,777	253	85,882	4,653	56.41	92.31
R1.2	81,638	78,105	19,768,333	253	73,531	4,669	57.00	92.12
R1.3	91,949	87,331	22,108,527	253	83,085	4,268	55.71	90.89
R2.1	85,402	80,429	20,352,629	253	60,140	3,815	58.24	91.01
R2.2	95,878	92,324	23,363,243	253	85,951	4,630	57.47	93.08

R2.3	94,049	89,408	22,721,237	254	81,009	4,344	58.07	90.82
R3.1	85,718	82,032	20,766,147	253	77,023	4,646	57.39	92.25
R3.2	88,747	85,270	21,590,636	253	79,761	4,512	57.55	92.71
R3.3	87,263	84,540	21,421,741	253	78,807	4,523	57.60	94.17
S1.1	80,036	76,511	19,396,376	254	71,693	4,733	57.68	91.98
S1.2	85,772	82,495	20,917,709	254	77,819	4,866	58.01	92.74
S1.3	82,830	78,714	20,106,433	255	68,673	4,939	57.76	91.53
S2.1	89,798	86,627	21,941,179	253	76,926	4,905	57.95	94.29
S2.2	96,001	92,863	23,517,539	253	82,667	5,407	57.76	94.50
S2.3	92,379	88,273	22,332,438	253	81,630	4,784	57.44	92.26
S3.1	91,021	87,786	22,300,570	254	78,748	4,477	58.38	94.23
S3.2	82,300	78,975	19,992,037	253	73,022	4,698	57.87	92.46
S3.3	85,723	82,103	20,772,979	253	76,356	4,450	57.97	92.34
Average	87,437	83,661	21,199,980	253	76,585	4,546	57.58	92.34

Legend: E, R and S represent the rhizosphere region of the top, middle and bottom of the slope respectively; 1, 2 and 3 in the middle represent 0-10cm, 10-25cm, and 25-40cm depths, respectively; 1, 2 and 3 in the end represent the first, second, and third repetitions, respectively.

Table S2 Variance analysis based on the relative abundance (100%) of the dominant bacteria in each sample.

		Pro	Cya	Act	Fir	Bac	Aci	Gem	Tha	Pla	Ver
	E	16.88±3.32b	1.86±1.47b	22.03±2.48a	0.32±0.03b	9.66±1.10ab	19.19±4.10	9.85±0.65b	0.50±0.15b	5.54±1.38	5.59±1.74a
0-10cm	R	22.87±4.65a	5.10±2.65a	18.09±2.97b	1.00±0.60a	11.16±2.30a	16.32±2.65	7.05±0.46c	0.64±0.10ab	5.55±2.09	4.08±0.43ab
	S	17.94±2.57b	0.73±0.70b	25.18±3.53a	0.45±0.24b	6.36±0.96b	17.94±4.62	13.97±1.05a	1.40±0.56a	3.73±0.90	2.85±0.58b
	E	15.77±1.74a	1.59±1.10b	21.71±2.23b	0.54±0.27c	4.79±0.37	18.37±1.08	16.71±0.89a	0.73±0.13b	2.37±0.51	4.76±0.18b
10-25cm	R	11.18±0.37b	2.98±1.24a	31.60±5.48a	0.33±0.18b	3.17±1.09	17.74±1.06	9.81±1.07b	1.03±0.38ab	5.23±1.07	6.86±1.20a
	S	16.50±0.71a	0.93±0.19c	28.97±1.68ab	0.79±0.42a	3.86±2.61	18.23±1.78	9.53±0.40b	2.00±0.72a	4.56±0.73	4.51±0.41b
	E	15.10±2.86	20.91±14.54a	19.14±5.37b	0.92±0.42a	3.17±1.03c	10.75±5.54b	15.74±7.28a	0.66±0.52b	3.34±1.52	2.31±0.08
25-40cm	R	15.99±1.14	0.67±0.38b	30.56±3.55a	0.42±0.17b	6.11±1.18a	19.46±1.56a	8.04±0.68b	1.55±0.33ab	4.63±0.43	3.85±0.83
	S	17.00±1.58	0.49±0.39b	32.53±2.95a	0.29±0.18c	4.01±1.91b	14.11±3.80b	13.20±2.10a	2.44±1.00a	3.25±1.23	2.92±0.86

Legend: E, R and S represent the top, middle and bottom of slope respectively; 1, 2 and 3 represent soil depths of 0-10cm, 10-25cm and 25-40cm respectively; Abbreviations: Pro, Proteobacteria; Cya, Cyanobacteria; Act, Actinobacteria; Fir, Firmicutes; Bac, Bacteroidetes; Aci, Acidobacteria; Gem, Gemmatimonadetes; Tha, Thaumarchaeota; Pla, Planctomycetes; Ver, Verrucomicrobia.

Table S3 Distribution of soil physical and chemical components (± SD; N = 3) at different slope positions and soil depths.

**Sample**	**E**	**R**	**S**	**1**	**2**	**3**
TOC	31.57±8.15a	23.72±3.97b	31.39±6.89a	26.56±4.56	29.76±9.84	30.38±6.92
TN	2.04±0.21	1.80±0.11	2.04±0.32	1.91±0.20	1.96±0.29	2.02±0.26
TP	0.84±0.41a	0.49±0.17b	0.41±0.16b	0.64±0.25	0.53±0.36	0.57±0.37
TK	17.74±1.58a	15.67±1.52b	16.89±1.68ab	17.61±1.98	16.19±1.82	16.50±1.24
NN	5.88±3.97b	5.22±1.89b	13.19±3.36a	7.56±2.88	8.33±5.76	8.39±5.72
AN	15.20±1.33b	16.63±1.69a	17.00±1.80a	15.92±2.09	16.88±1.89	16.03±1.61
AP	7.72±3.70a	2.69±1.3.a	3.76±1.72a	6.52±4.46a	3.87±2.31b	3.79±1.87b
TS	8.70±6.33a	2.85±1.69a	8.55±6.56a	1.93±1.52b	7.34±6.49a	10.83±5.66a
pH	7.84±0.26	7.91±0.13	8.22±0.77	8.25±0.46a	7.97±0.50ab	7.75±0.41b

Legend: E, R and S represent the top, middle and bottom of slope respectively; 1, 2 and 3 represent soil depths of 0-10cm, 10-25cm and 25-40cm respectively; TS, total salt content; AP, available phosphorus content; AN, ammonium nitrogen content; NN, nitrate nitrogen content; TK, total potassium content; TP, total phosphorus content; TN, total nitrogen content; TOC, total organic carbon content; SD, standard deviation.

Table S4 The results for db-RDA testing effects of soil properties on the composition of rhizosphere bacterial communities across all sampling sites.

**Variables**	**% variance explained**	***p*-value**
All Factors	24.28	0.004
pH	5.58	0.002
TS	5.21	0.002
TP	4.90	0.006
NN	3.89	0.004
AP	3.60	0.006
AN	3.19	0.036
TOC	2.83	0.005
TN	2.48	0.026
TK	2.22	0.024

Legend: TS, total salt content; AP, available phosphorus content; AN, ammonium nitrogen content; NN, nitrate nitrogen content; TK, total potassium content; TP, total phosphorus content; TN, total nitrogen content; TOC, total organic carbon content.

Table S5 *Ferula sinkiangesis* mixed labeling method for all soil samples.

	Sample	Label	Label order
Rhizosphere slope positions	Top	E	1
	Middle	R	
	Bottom	S	
Soil depth	0-10cm	1	2
	10-25cm	2	
	25-40cm	3	
Repetition	First repeat	1	3
	Second repeat	2	
	Third repeat	3	

Table S6 Statistics of physicochemical properties (± SD; N = 3) of all soil samples.

Sample	TOC	TN	TP	TK	NN	AN	AP	TS	pH
E1.1	25.75	2.12	0.40	20.56	7.23	14.52	12.59	1.24	8.08
E1.2	30.81	1.98	1.01	19.09	5.60	15.13	8.81	1.11	8.33
E1.3	30.49	2.05	1.07	18.11	5.41	12.85	14.49	0.95	7.98
E1	29.02±2.83	2.05±0.07	0.83±0.37	19.25±1.23	6.08±1.00	14.17±1.18	11.97±2.89	1.10±0.14	8.13±0.18
E2.1	18.22	1.67	0.19	18.68	2.66	17.57	2.62	7.10	7.62
E2.2	43.55	2.01	0.97	17.47	2.30	15.05	6.41	14.78	7.75
E2.3	39.28	2.42	1.17	15.70	14.11	14.56	6.67	11.59	7.87
E2	33.68±13.56	2.03±0.38	0.78±0.52	17.28±1.50	6.35±3.41	15.73±1.61	5.24±2.21	11.15±3.86	7.75±0.13
E3.1	23.30	1.91	0.33	16.98	3.06	16.40	6.23	11.17	7.46
E3.2	36.83	2.02	1.26	17.39	2.73	15.91	5.30	17.45	7.79
E3.3	35.92	2.21	1.14	15.67	9.81	14.79	6.36	12.88	7.69
E3	33.02±7.56	2.05±0.15	0.91±0.51	16.68±0.90	5.20±3.99	15.70±0.83	5.96±0.58	13.83±3.25	7.65±0.17
R1.1	26.72	1.87	0.64	16.14	5.27	19.95	5.58	1.15	8.05
R1.2	21.25	1.67	0.60	15.53	5.39	15.73	2.53	1.00	7.92
R1.3	21.01	1.58	0.54	17.21	5.56	18.06	3.84	1.20	7.84

R1	22.99±3.23	1.71±0.15	0.59±0.24	16.29±0.85	5.41±0.15	17.91±2.11	3.98±1.53	1.12±0.11	7.94±0.10
R2.1	20.05	1.88	0.69	15.43	1.65	15.67	1.68	0.58	8.02
R2.2	20.72	1.83	0.48	15.26	7.53	17.14	1.72	0.76	7.98
R2.3	28.98	1.80	0.14	13.29	5.06	15.93	2.77	1.15	8.01
R2	23.25±4.98	1.83±0.37	0.44±0.28	14.67±1.19	4.75±2.95	16.24±0.78	2.06±0.62	0.83±0.29	8.01±0.02
R3.1	20.16	1.78	0.51	14.79	4.03	17.01	1.48	1.16	7.94
R3.2	30.03	1.91	0.32	14.81	8.09	14.01	2.21	9.95	7.74
R3.3	24.58	1.88	0.46	18.60	4.41	16.21	2.42	8.71	7.68
R3	24.90±4.94	1.86±0.65	0.43±0.27	16.06±0.27	5.51±2.25	15.74±1.55	2.04±0.49	6.61±4.76	7.79±0.14
S1.1	21.20	1.84	0.59	14.37	12.71	14.74	2.93	4.80	8.36
S1.2	29.03	1.88	0.43	19.29	9.86	15.63	5.17	4.39	8.29
S1.3	32.73	2.21	0.42	18.23	11.02	16.63	2.69	1.56	9.37
S1	27.66±5.89	1.98±0.20	0.48±0.09	17.29±2.58	11.20±1.43	15.67±0.94	3.60±1.37	3.58±1.17	8.67±0.60
S2.1	26.22	1.89	0.59	18.34	15.72	19.06	2.85	16.20	8.01
S2.2	28.07	1.71	0.31	14.41	15.13	16.64	2.55	12.34	7.33
S2.3	42.73	2.45	0.21	17.09	10.85	20.35	7.53	1.55	9.16
S2	32.34±9.04	2.02±0.38	0.37±0.19	16.62±2.00	13.90±2.66	18.68±1.88	4.31±2.17	10.03±7.59	8.17±0.93

S3.1	29.54	1.84	0.60	16.98	14.68	15.26	2.25	16.46	7.46
S3.2	31.66	1.94	0.20	16.37	9.20	17.38	3.15	16.01	8.70
S3.3	41.34	2.64	0.32	16.91	19.55	17.30	4.69	3.68	7.31
S3	34.18±6.29	2.14±0.43	0.38±0.21	16.75±0.33	14.48±5.18	16.65±1.20	3.37±1.23	12.05±7.26	7.82±0.76

Legend: E, R and S represent the rhizosphere region of the top, middle and bottom of the slope respectively; 1, 2 and 3 in the middle represent 0-10cm, 10-25cm, and 25-40cm depths, respectively; 1, 2 and 3 in the end represent the first, second, and third repetitions, respectively; TS, total salt content; AP, available phosphorus content; AN, ammonium nitrogen content; NN, nitrate nitrogen content; TK, total potassium content; TP, total phosphorus content; TN, total nitrogen content; TOC, total organic carbon content; SD, standard deviation.

Figure S1 Rarefaction curves of OTUs at 97% similarity for each sample.

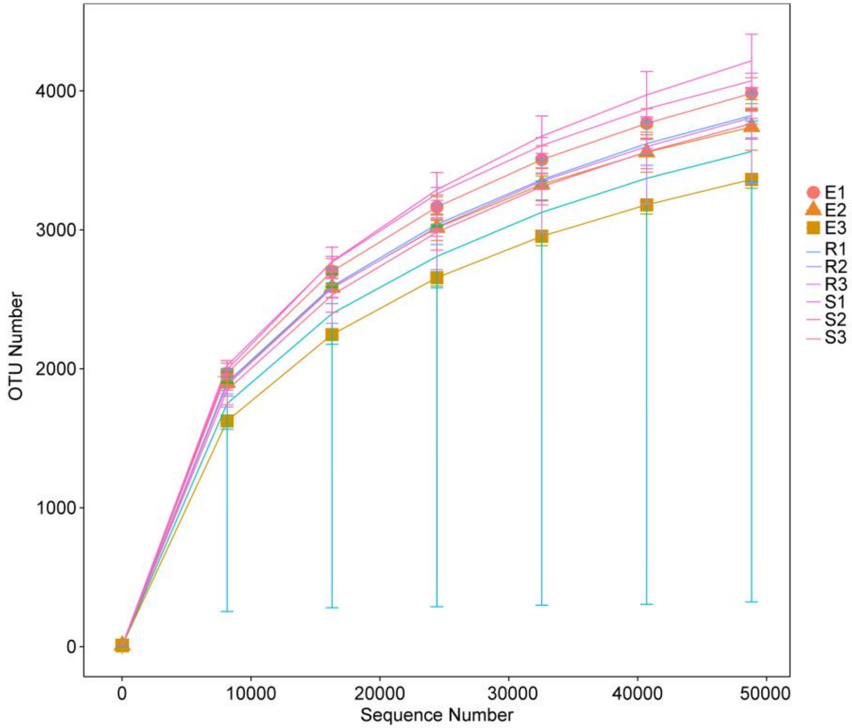

Legend: Longitudinal direction isthe number of OTUs constructed based on the number of sequencing strips, the horizontal direction is thenumber of sequencing strips randomly extracted from a sample. Different samples are represented by curves with different colors.

Figure S2 The relative abundance of the top 35 genera.

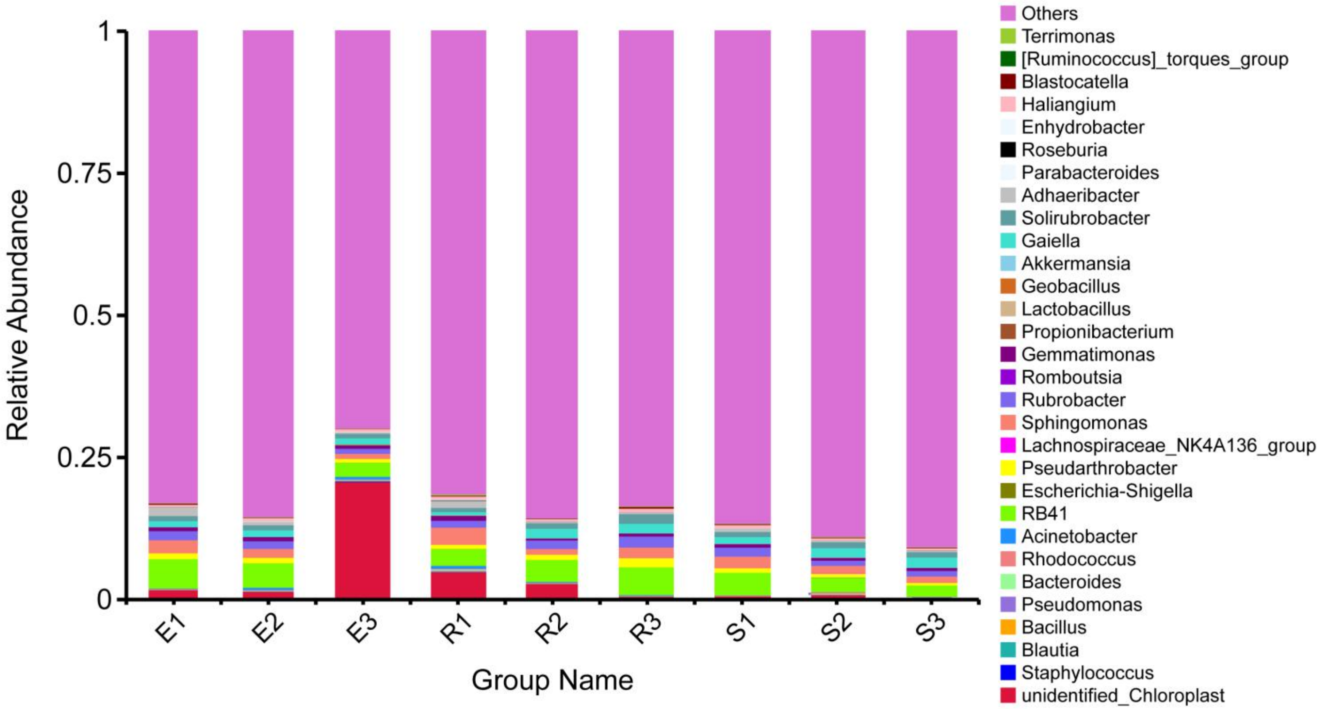

Legend: The abscissa is the sample name; the ordinate indicates the relative abundance; Others indicates genera outside the top 30 of the annotated list and the parts not annotated.

FigureS3 Spearman rank-related heat map shows the relationship between environmental factors and bacterial phyla level richness (α-diversity).

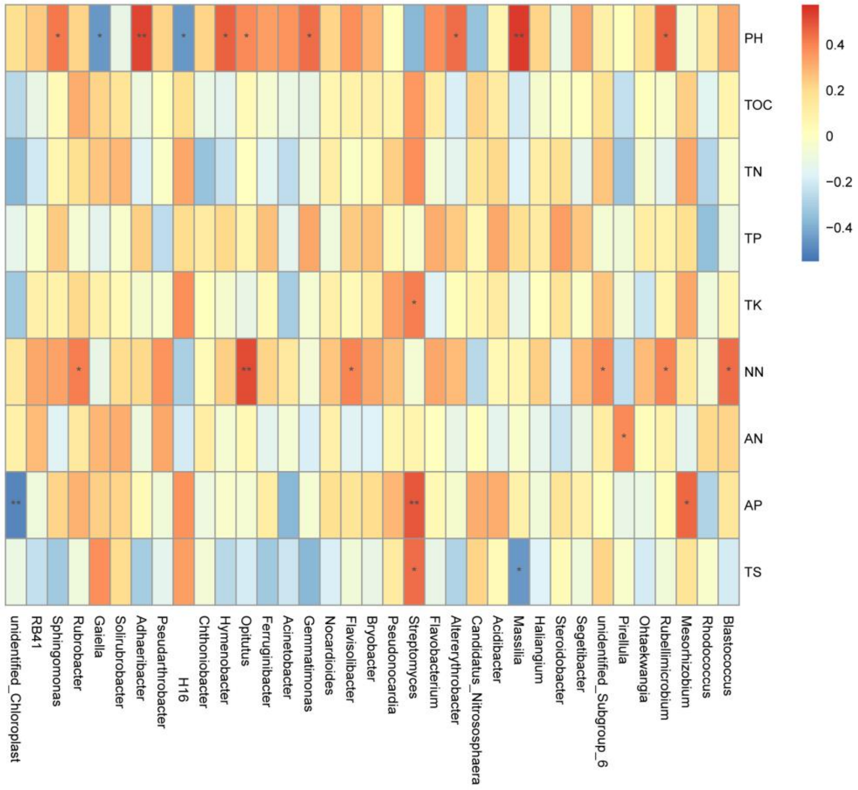

Legend: Longitudinal direction is the environmental factor information, the horizontal direction is the species information, and the intermediate heat map corresponds to the Spearman correlation coefficient r, which is between -1 and 1, r<0 is a negative correlation, r>0 is a positive correlation $*p < 0.05$, $**p < 0.01$.
